# Chemopreventative celecoxib fails to prevent schwannoma formation or sensorineural hearing loss in genetically engineered murine model of neurofibromatosis type 2

**DOI:** 10.18632/oncotarget.22002

**Published:** 2017-10-24

**Authors:** Benjamin M. Wahle, Eric T. Hawley, Yongzheng He, Abbi E. Smith, Jin Yuan, Andi R. Masters, David R. Jones, Jeffrey R. Gehlhausen, Su-Jung Park, Simon J. Conway, D. Wade Clapp, Charles W. Yates

**Affiliations:** ^1^ Wells Center for Pediatric Research, Department of Pediatrics, Indiana University School of Medicine, Indianapolis, Indiana, USA; ^2^ Department of Otolaryngology/Head and Neck Surgery, Indiana University School of Medicine, Indianapolis, Indiana, USA; ^3^ Clinical Pharmacology Analytical Core, Indiana University Simon Cancer Center, Indianapolis, Indiana, USA

**Keywords:** neurofibromatosis type 2, vestibular schwannoma, cyclooxygenase 2, non-steroidal anti-inflammatory agents, transgenic mice

## Abstract

Mutations in the tumor suppressor gene *NF2* lead to Neurofibromatosis type 2 (NF2), a tumor predisposition syndrome characterized by the development of schwannomas, including bilateral vestibular schwannomas with complete penetrance. Recent work has implicated the importance of COX-2 in schwannoma growth. Using a genetically engineered murine model of NF2, we demonstrate that selective inhibition of COX-2 with celecoxib fails to prevent the spontaneous development of schwannomas or sensorineural hearing loss *in vivo*, despite elevated expression levels of COX-2 in *Nf2*-deficient tumor tissue. These results suggest that COX-2 is nonessential to schwannomagenesis and that the proposed tumor suppressive effects of NSAIDs on schwannomas may occur through COX-2 independent mechanisms.

## INTRODUCTION

Neurofibromatosis type 2 (NF2) is an inherited tumor predisposition syndrome affecting 1 in 25,000 people, most of whom are germline heterozygous for the *NF2* tumor suppressor gene [1]. The hallmark of NF2 is the development of bilateral vestibular schwannomas (VS), which lead to sensorineural hearing loss, tinnitus, and vestibular dysfunction as well as brainstem compression, hydrocephalus, and death with sufficiently large tumors. There are currently no approved chemotherapeutic agents for the treatment of schwannomas, owing in part to an incomplete understanding of the biochemical derangements that occur as a result of *NF2*-deficiency.

Although the molecular pathogenesis of VS is an area of active investigation, several lines of evidence support the hypothesis that COX-2 overexpression contributes to schwannoma growth. COX-2 is an inducible enzyme involved in the biosynthesis of prostanoids from arachidonic acid. Prostaglandin E_2_ (PGE_2_) is the best-understood effector of COX-2 in the setting of malignancy and has been implicated both as having direct effects on tumor cells as well as affecting immune surveillance within the tumor microenvironment [2]. Immunohistochemical staining of spontaneous and NF2-related VS suggested a correlation between tumor proliferation and COX-2 expression [3]. A retrospective case series found that low dose aspirin inversely correlated with the propensity for sporadic VS growth on serial magnetic resonance imaging. This finding suggests that there may be a link between aspirin’s COX-2 or NFκB inhibitory functions and schwannoma growth [4]. Importantly, compared to normal Schwann cells, primary cultured human VS cells were shown to have increased COX-2 expression, increased secretion of PGE_2_, and reduced proliferation when treated with a COX-2 inhibitor [5].

COX-2 also intersects with a second biochemical pathway of recent interest in NF2 pathogenesis. The mammalian Hippo pathway has been a source of great interest in the development of NF2-deficient schwannomas. Guerrant and colleagues recently found that COX-2 is transcriptionally up regulated by YAP, a Hippo pathway effector that is transcriptionally activated in the setting of *NF2-* deficiency [6]. Further, that same group demonstrated that the COX-2 inhibitor celecoxib suppressed tumor expansion in an orthotopic model of tumorigenesis [6]. Given that recent data, we questioned whether COX-2 inhibition could be used as a chemopreventative strategy in NF2 mediated schwannoma formation, especially in light of the success of inhibition of COX-2 in other cancer predisposition syndromes [7]. In addition to its value as a treatment modality, effective chemoprevention would provide a rationale for diagnosing individuals at known genetic risk for NF2 prior to tumor initiation.

While no animal model system can serve as a perfect analog to human disease, there are unique advantages to the use of genetically engineered mouse (GEM) models of cancer. Compared to cell-line derived xenograft (CDX) models, GEM cancer models provide a much closer surrogate for human disease by allowing the spontaneous development of tumors from defined primary tumorigenic cells within their native stroma and immune microenvironment [8]. The *Nf2*^*flox/flox*^*;Periostin-Cre (Nf2*^*f/f*^*;PostnCre)* mouse model developed in our laboratory eliminates the production of Merlin, the protein product of *Nf2*, in the Schwann cell lineage. Mice expressing Cre recombinase develop spontaneous schwannomas of the dorsal root ganglia (DRG) and cranial nerves with 100% penetrance [9]. Using this established model of NF2, we tested the hypothesis that chemopreventative COX-2 inhibition attenuates spontaneous schwannomagenesis or sensorineural hearing loss (SNHL).

## RESULTS

### COX-2 is overexpressed in *Nf2*-deficient mouse model of NF2

**Figure 1:**
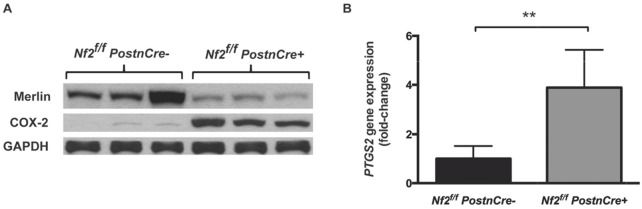
COX-2 is increased in *Nf2*-deficient mouse model of neurofibromatosis type 2 **(A)** Western blot analysis of COX-2 and Merlin in whole tissue lysate of tumor-forming trigeminal ganglia tissue of 6-month old *Nf2*^*f/f*^*;PostnCre-* or *Nf2*^*f/f*^*;PostnCre+* mice. **(B)** Relative *PTGS2* gene expression in trigeminal ganglia tissue of 6-8 month old *Nf2*^*f/f*^*;PostnCre-* or *Nf2*^*f/f*^*;PostnCre+* mice by qPCR. (^**^ *P*=.004).

To assess whether COX-2 was overexpressed in the setting of *Nf2-*deficient tumor-forming tissue, we examined protein levels of COX-2 in the trigeminal ganglia of 6-month-old mice. At this anatomic site, mice with a conditional-knockout of *Nf2* (*Nf2*^*f/f*^*;PostnCre+*) exhibit Schwann cell hyperplasia or frank schwannoma formation by 6 months of age. Relative to strain equivalent *Nf2*-sufficient mice (*Nf2*^*f/f*^*;PostnCre-)*, COX-2 was markedly increased in the trigeminal ganglia of *Nf2*^*f/f*^*;PostnCre+* mice (Figure 1A). As expected, levels of the *Nf2* protein product Merlin were diminished in the *Nf2*^*f/f*^*;PostnCre+* tissue relative to *Nf2-*sufficient controls. The faint Merlin immunostaining present in the *Nf2*^*f/f*^*;PostnCre+* samples likely represents Merlin expression in non-Schwann cell lineages within the ganglia.

We then examined the expression of *PTGS2*, the gene encoding COX-2, in RNA extracted from trigeminal ganglia tissue. *PTGS2* expression was increased 3.9-fold in *Nf2*^*f/f*^*;PostnCre+* mice (95% CI 1.97–5.80) compared to *Nf2*^*f/f*^*;PostnCre-* controls (*P=*.004, Figure 1B). This fold-increase in expression is comparable to that observed by Dilwali *et al.* in primary cultured human VS cells [5]. To better approximate the *PTGS2* fold-increase, one tissue sample from an *Nf2*^*f/f*^*;PostnCre+* mouse was removed as it met the criteria for a high outlier (*PTGS2* fold-increase of 16.0). Together, these results suggest that COX-2 is aberrantly expressed in *Nf2-*deficient, tumor-forming tissue, a finding consistent with prior published work [5].

### Chemopreventative celecoxib treatment of *Nf2*-deficient mice

**Figure 2:**
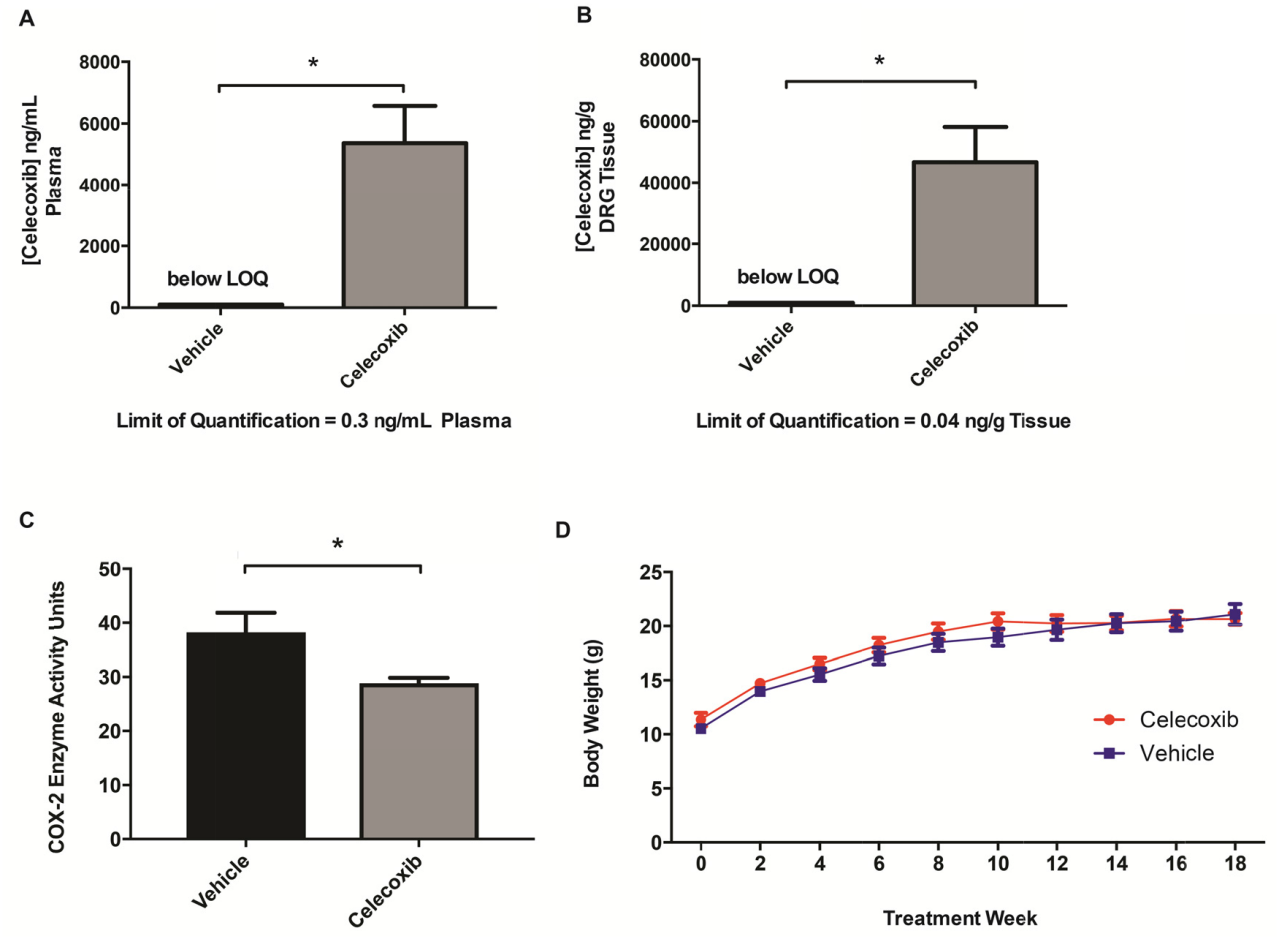
Chemopreventative celecoxib treatment of *Nf2-*deficient mice HPLC-MS/MS analysis of celecoxib levels in **(A)** plasma and **(B)** DRG tissue between treated and untreated mice. (^*^P <.05) **(C)** Mice were fed vehicle or celecoxib infused chow for 5 days followed by an IP injection of 100ng of LPS for induction of COX-2. Mice were sacrificed 2 hours post injection. Y axis measures COX-2 activity in units/mL of tissue lysate (P= 0.0379, unpaired t-test). **(D)** Mouse body weights of treated and untreated *Nf2*^*f/f*^*;PostnCre+* mice.

To test the hypothesis that pharmacologic COX-2 inhibition prevents or slows schwannoma growth, we performed a chemopreventative trial of celecoxib in *Nf2*^*f/f*^*;PostnCre+* mice beginning at 3-5 weeks of age, before the initiation of Schwann cell hyperplasia or frank schwannoma formation. Celecoxib was administered through an *ad libitum* drug-containing diet until the mice reached six months of age. Celecoxib’s half-life has been reported to be as low as two hours in mice [10]. In addition to being safer than gavage feeding drug multiple times per day for several months, administration of celecoxib through an *ad libitum* diet made animals more likely to achieve relatively constant levels of celecoxib. Once a celecoxib diet was formulated, pharmacokinetic evaluation in the plasma and relevant peripheral nervous system tissue was performed. We performed HPLC-MS/MS on plasma and DRG samples of *Nf2*^*f/f*^*;PostnCre-* mice that had been fed celecoxib (N=4) or vehicle diets (N=3) for one week. Substantial plasma celecoxib levels were achieved in all treated mice (Figure 2A; mean 5361 ng/mL plasma; range 2883 to 8230 ng/mL). These plasma values, which likely represent steady-state celecoxib levels in our animals, are on the order of maximum concentrations achieved with high-dose celecoxib treatment in humans [11]. Similar plasma concentrations were achieved in a previous study using a GEM cancer model that responded to an *ad libitum* celecoxib diet [12]. We also confirmed that celecoxib effectively accumulated in the DRG, our therapeutic target (Figure 2B; mean 46672 ng/g tissue; range 24390 to 76086 ng/g). For mice consuming vehicle diet, no celecoxib was detected in plasma or DRG samples.

To ensure our celecoxib-containing diet effectively inhibited murine COX-2 *in vivo* using our dosing strategy, mice were fed either the celecoxib-containing or control diet for five days and were then injected with 100ng lipopolysaccharide (LPS) intraperitoneally to induce COX-2 expression. LPS has previously been demonstrated to strongly induce COX-2 protein levels and downregulate COX-1 in astrocytes [13]. Because levels of COX-2 are among the highest in brain tissue, the right cerebral hemisphere was harvested two hours after injection. Mice fed the celecoxib infused diet had a significant reduction in COX-2 activity in nervous tissue following induction and activation of COX-2 (Figure 2C). This demonstrates that the tissue concentrations of celecoxib achievable via free feeding are sufficient for significant biochemical suppression of COX-2 in our murine model. Taken together, these experiments indicate that treatment with *ad libitum* celecoxib diet effectively inhibits COX-2 and results in drug concentrations generally exceeding those achieved in humans.

### Celecoxib does not prevent schwannomagenesis in *Nf2*-deficient mice

Animals began celecoxib treatment at 3-5 weeks of age and were treated to the age of six months. Celecoxib treatment was generally well tolerated and mice exhibited normal growth compared to those on vehicle diet (Figure 2D). Three of twelve celecoxib mice and one of thirteen vehicle mice died prior to six months of age, a difference in survival that was not statistically significant (*P*=.30, survival curves not shown), suggesting that chemopreventative celecoxib therapy does not improve survival in our model.

**Figure 3:**
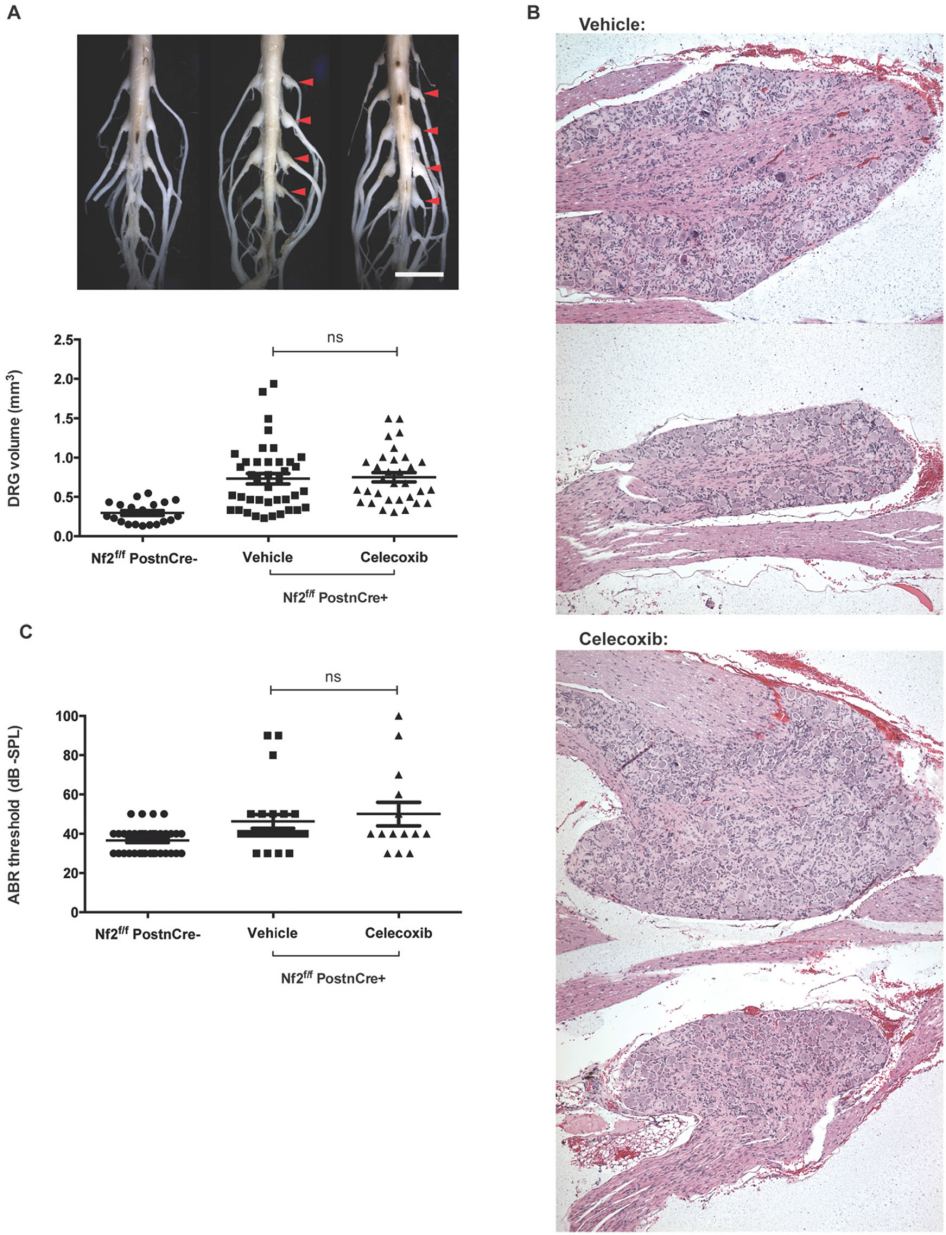
Celecoxib fails to prevent schwannomagenesis or SNHL in *Nf2*-deficient mice **(A)** DRG enlargement – Representative images of gross DRG enlargement between a *Nf2*^*f/f*^*;PostnCre-* mouse (left), vehicle and celecoxib-treated *Nf2*^*f/f*^*;PostnCre+* mice (center and right) are shown (scale bar = 5 mm). Below, DRG volume is shown for the same groups of mice (ns = not significant; *P* >.05). **(B)** DRG histology – Schwannomas at later and earlier stages of development are shown for both vehicle and celecoxib-treated mice. **(C)** ABR – Thresholds are shown per ear for *Nf2*^*f/f*^*;PostnCre-*, vehicle and celecoxib-treated *Nf2*^*f/f*^*;PostnCre+* mice.

*Nf2*^*f/f*^*;PostnCre+* mice spontaneously develop schwannomas between 5-8 months of age [9]. The endpoint of six months was chosen because this is a time point at which a delay in schwannoma growth would most likely be detected as a difference in tumor volume and number. By six months, both celecoxib and vehicle treated *Nf2*^*f/f*^*;PostnCre+* mice had gross enlargements of DRG compared to *Nf2*^*f/f*^*;PostnCre-* mice (Figure 3A). DRG volume was significantly increased in both celecoxib and vehicle treated mice relative to *Nf2*-sufficient controls (adjusted *P*<.001 in both cases). No difference in tumor volume was present between celecoxib and vehicle groups (adjusted *P*=.97).

To determine whether the rate of DRG enlargement differed between groups, a threshold for pathologically increased DRG size was determined. Any DRG greater than the 95^th^ percentile of *Nf2*^*f/f*^*;PostnCre-* was considered to be enlarged. No significant difference in the rate of DRG enlargement was present between celecoxib and vehicle groups (69% and 55% respectively; *P*=.33).

DRG of celecoxib-treated mice had histologic features consistent with schwannoma and were indistinguishable from DRG of vehicle-treated controls (Figure 3B). DRG architecture was disrupted by Schwann cell proliferation to varying degrees within a single animal. Taken together, these data suggest that chemopreventative celecoxib does not delay spontaneous schwannoma formation or growth *in vivo*.

### Celecoxib does not prevent SNHL in *Nf2*-deficient mice

*Nf2*^*f/f*^*;PostnCre+* mice develop spontaneous SNHL which can be detected by changes in ABR threshold as previously described [9]. ABR testing was conducted at six months of age prior to sacrificing mice. No differences were present between thresholds of celecoxib and vehicle groups (adjusted *P*=.72). Thresholds of both vehicle and celecoxib-treated *Nf2*^*f/f*^*;PostnCre+* mice were significantly higher than those of an age-matched group of *Nf2*^*f/f*^*;PostnCre-* mice (adjusted *P*=.04 and.01 respectively), suggesting that hearing loss was present in this study’s treatment and control groups. We conclude that celecoxib treatment did not prevent SNHL in our murine model of NF2.

## DISCUSSION

Our findings fail to establish the efficacy of COX-2 inhibition as a strategy for the prevention of spontaneous tumorigenesis or SNHL in NF2. They support a different conclusion than the evidence on this question recently published by Guerrant *et al.*, who demonstrated that celecoxib inhibits tumor growing *in vivo* [6]. There are differences in design between the two studies. Rather than treating existing tumors we sought to determine whether we could prevent tumor initiation. Tumor development in the *Nf2*^*f/f*^*;PostnCre* model is spontaneously initiated by loss of *Nf2* in the Schwann cell lineage, and occurs over a period of months [9]. In contrast, tumors formed by Guerrant *et al.* were caused by implantation of the SC4 cell line in immunodeficient mice. SC4 cells are a rapidly growing, immortalized *Nf2*-deficient murine Schwann cell line. These cells have a doubling time between 24 and 48 hours and form tumors over a period of days [6]. As others have noted, the biologic relevance of CDX models can be diminished by processes related to 2D cell culture, including the immortalization of cells and selection that occurs over the course of several cell passages [8]. In our hands, other immortalized *NF2-* deficient lines such as HEI-193 have hyperproliferative phenotypes that are unchanged by Merlin re-expression. For this reason, therapeutic responses in such cell lines may represent responses of acquired oncogenic drivers not present in human disease. Additionally, the use of immunocompetent GEM models better approximates the tumor microenvironment, a detail of particular importance in the study of anti-inflammatory agents that may target microenvironmental cells [8].

Several other studies using GEM models of cancer have demonstrated therapeutic responses to celecoxib at lower or comparable doses than those used in our study [12, 14, 15]. We demonstrated that our celecoxib treatment achieved considerable plasma levels, effectively reached tumor-forming tissue, and effectively decreased COX-2 activity. Our mice were treated for several months throughout the period of spontaneous tumorigenesis. Although failing to demonstrate its therapeutic relevance, it is important to note that our mouse model recapitulated the aberrant COX-2 expression seen in Dilwali *et al’s* study comparing primary cultured human VS cells and normal human Schwann cells [5]. The use of this mouse model for *in vivo* validation of biochemical derangements identified in primary cultured human VS cells may represent an effective strategy for finding translationally relevant targets for the treatment of VS.

Work of multiple groups suggests that NSAIDs, particularly aspirin, may have therapeutic potential in VS [4, 5, 16]. While our findings do not preclude this possibility, they do suggest that a response to NSAIDs would occur through COX-2 independent mechanisms. Aspirin and its metabolite salicylate are also inhibitors of the NFκB pathway, which is also activated in VS [17–20]. This inhibition occurs at much lower doses than does aspirin’s inhibition of COX-2, suggesting the potential that NFκB inhibition may be aspirin’s more therapeutically relevant effect [21]. There are also other NSAIDs, such as diclofenac, which have shown promise in other cancers but remain untested in VS [22, 23]. Further study using effective cellular and animal models is needed to determine whether other molecular targets of NSAIDs are effective in the treatment of VS.

## MATERIALS AND METHODS

### Animals and celecoxib administration

The Indiana University Institutional Animal Care and Use Committees approved the animal protocol (#10940). *Nf2*^*flox/flox*^*;Periostin-Cre* mice have been previously described [9]. Mice were fed a diet formulated to deliver 300 mg/kg/day of celecoxib or an otherwise identical vehicle diet (ResearchDiets). Mouse weights were recorded every two weeks. Mice were sacrificed at 6 months of age and four DRG tumors at consistent anatomic locations were measured per mouse as previously described [9].

### Protein extraction and western blot

Trigeminal ganglia were dissected from 6-month-old mice and washed thoroughly in PBS. Protein was isolated in cell lysis buffer with protease and phosphatase inhibitors. Protein concentrations were measured using a bicinchoninic acid assay (Thermo Scientific) and normalized. Protein samples were resolved with SDS-polyacrylamide gel electrophoresis and transferred onto polyvinylidene difluoride membranes. COX-2 antibody was purchased from Santa Cruz Technologies and antibodies for Merlin and GAPDH were purchased from Cell Signaling Technologies.

### Reverse transcription-quantitative polymerase chain reaction

RNA was extracted from trigeminal ganglia of 6-8 month old mice using Trizol Reagent (Life Technologies) per the manufacturer’s instructions. RNA concentration was determined using Nanodrop (Thermo Scientific) and converted to cDNA using a QuantiTect reverse transcription kit (Qiagen). Quantitative PCR was performed using a SYBR green kit (Life Technologies), probes for PTGS2 and GAPDH, and an Applied Biosystems 7500 System.

### High performance liquid chromatography and mass spectrometry

High performance liquid chromatography (HPLC) and Mass Spectrometry (MS) were performed by the IU Simon Cancer Center’s Clinical Pharmacology Analytical Core. Samples were acidified and extracted in hexane:ethyl acetate (50:50, v/v). After solvent evaporation, mobile phase (acetonitrile:5mM ammonium acetate; 70:30, v/v) was mixed with residual sample and injected into an Agilent 1290 HPLC system with an Eskigent Autosampler. Mass spectrometry was performed using an ABSciex 5500 Q-TRAP.

### COX-2 activity ELISA

8-month-old *Nf2*^*f/f*^*;PostnCre-* mice were separated into two groups of 5 mice and fed either celecoxib or vehicle diet for 5 days. On day 5, the mice were injected intraperitoneally with 100ng of tissue culture grade *Escherichia coli* O111:B4 derived LPS in 100ul of sterile PBS. Mice were sacrificed 2 hours later. The left cerebral hemisphere was harvested from each mouse. Tissues were processed and samples were run as described following the factory protocol using the Cayman Chemical COX Activity Assay Kit (760151).

### Auditory brain stem response

Prior to harvest, mice were anesthetized with 100 mg/kg ketamine and 10 mg/kg xylazine by intraperitoneal injection. ABR signals were recorded from each ear using a Tucker Davis Technologies (TDT) MF1 speaker and subcutaneous needle electrodes connected to a TDT RZ6 Multi I/O processor. Clicks were presented at 21/s with 512 repetitions, sweeping from 30 to 90 dB-SPL in 10 dB intervals. Signals were high and low pass filtered (3 Hz-3 kHz) and averaged in TDT BioSigRZ. Thresholds were defined as the lowest SPL at which more than one peak was observed and reproducible.

### Statistical analysis

qPCR and mouse weights were compared using unpaired t tests. For DRG volume and ABR threshold data, one-way ANOVA was used and Tukey’s posttest was performed to generate adjusted *P* values. Chi-square test was used to test differences in rate of DRG enlargement. Survival between groups was compared using a log-rank test. Grubbs test for statistical outliers was performed for all data sets. *P*<.05 was considered statistically significant for all tests.

## Abbreviations

COX-2: (cyclooxygenase-2)
NSAID: (nonsteroidal antiinflamatory drug)
